# Barriers to Prompt Presentation to Emergency Departments in Colorado after Onset of Stroke Symptoms

**DOI:** 10.5811/westjem.2018.10.38731

**Published:** 2018-12-05

**Authors:** Stacy A. Trent, Erica A. Morse, Adit A. Ginde, Edward P. Havranek, Jason S. Haukoos

**Affiliations:** * Denver Health Medical Center, Department of Emergency Medicine, Denver, Colorado; † University of Colorado School of Medicine, Department of Emergency Medicine, Aurora, Colorado; ‡ St. Joseph’s Hospital, Department of Emergency Medicine, Denver, Colorado; § Colorado School of Public Health, Department of Epidemiology, Aurora, Colorado; ¶ Denver Health Medical Center, Department of Medicine, Denver, Colorado; || University of Colorado School of Medicine, Division of Cardiology, Aurora, Colorado

## Abstract

**Introduction:**

Despite significant morbidity and mortality from stroke, patient delays to emergency department (ED) presentation following the onset of stroke symptoms are one of the main contraindications to treatment for acute ischemic stroke (AIS). Our objective was to identify patient and environmental factors associated with delayed presentations to the ED after onset of stroke symptoms.

**Methods:**

This was a pre-planned secondary analysis of data from a multicenter, retrospective observational study at three hospitals in Colorado. We included consecutive adult patients if they were admitted to the hospital from the ED, and the ED diagnosed or initiated treatment for AIS. Patients were excluded if they were transferred from another hospital. Primary outcome was delayed presentation to the ED (> 3.5 hours) following onset stroke symptoms.

**Results:**

Among 351 patients, 63% presented to the ED more than 3.5 hours after onset of stroke symptoms. Adjusted results show that patients who presented in the evening hours (odds ratio [OR] [0.45], 95% confidence interval [CI] [0.3–0.8]), as compared to daytime, were significantly less likely to have a delayed presentation. Speaking a language other than English (Spanish [OR 3.3, 95% CI 1.2–8.9] and “other” [OR 9.1, 95% CI 1.2–71.0]), having known cerebrovascular risk factors (>2 risk factors [OR 2.4, 95% CI 1.05–5.4] and 1–2 risk factors [OR 2.3, 95% CI 1.03–5.1], compared to zero risk factors), and presenting to a rural hospital (OR 2.2, 95% CI 1.2–4.2), compared to urban, were significantly associated with delayed presentation.

**Conclusion:**

Important patient and environmental factors are significantly associated with delayed ED presentations following the onset of stroke symptoms. Identifying how best to educate patients on stroke risk and recognition remains critically important.

## INTRODUCTION

Cerebrovascular disease is the fourth leading cause of death in the United States (U.S.)^1^ For patients who survive a stroke, daily functionality may be permanently affected resulting in severe disability.^2^ Intravenous thrombolysis using tissue plasminogen activator (tPA) has the potential to improve morbidity in patients who present to an emergency department (ED) shortly after the onset of symptoms.^3^ Despite significant morbidity and mortality from stroke, patient delays to ED presentation following the onset of stroke symptoms continue to be the main contraindication to using tPA for acute ischemic stroke (AIS).^4^ Our prior work examining variation in adherence to guideline recommendations for administration of tPA for AIS identified that most patients were not eligible for tPA because they presented to the ED well outside the recommended treatment window. Thus, our objective was to identify patient and environmental factors that may contribute to delays in presentation following the onset of stroke symptoms in our patient population.

## METHODS

### Study Design

We conducted a pre-planned, secondary analysis of data from a multicenter, retrospective observational study evaluating variation in ED adherence to cardiovascular and cerebrovascular guidelines, including systemic thrombolysis for AIS.^5^ The institutional review boards at each participating hospital approved the study with a waiver of consent.

### Setting

This study was performed at three acute care hospitals in Colorado, including an urban safety-net hospital, a suburban, academic tertiary-care hospital, and a rural community hospital. All three EDs were staffed by board-certified/eligible emergency physicians. Annual adult ED census ranged from 55,000 to 80,000 patients at each hospital. Only the academic tertiary-care hospital was a certified Joint Commission Stroke Center. The rural community ED had neurologists available for consultation only by video, whereas the two other EDs had 24/7 in-house neurology consultation.

### Population and Assembly of the Study Cohort

Consecutive patients were identified retrospectively by any hospital-discharge implantable cardioverter-defibrillator ICD-9 code for acute ischemic stroke (434.xx).^6^ Investigators at each site obtained a list of consecutive patients with the above ICD-9 codes, who were admitted from the ED beginning on December 31, 2012. From this initial cohort, each unique patient encounter was screened by a physician abstractor for inclusion using the following criteria: 1) a discharge diagnosis in the medical record of acute ischemic stroke; 2) admission to the hospital from the ED; and 3) diagnosis or initiated treatment for AIS in the ED. Exclusion criteria were age <18 years and patients transferred from another facility. Patient encounters were screened until we obtained a sufficient sample (n=117) at each institution.

### Data Collection

Once the study cohort was established, structured medical record abstraction was performed using established, standard methodology.^7^,^8^ Using a structured data abstraction form, abstractors documented the presence of pre-specified variables necessary to assess guideline adherence for tPA use in AIS, including time of symptom onset defined as last known normal (Appendix). In addition, patient sociodemographics, cerebrovascular comorbidities, stroke symptoms, arrival day and time were collected.^9^–^12^ We stratified patients’ stroke risk into one of three groups depending on the cumulative number of stroke comorbidities: none, 1–2, and > 2. Patient chief complaints were stratified into three groups related to how typical the complaint was for stroke: typical for stroke (focal weakness, numbness, or alteration in speech); associated with stroke (headache, ataxia, dizziness, fall, seizure, vision change, altered mental status); and other.

**Population Health Research Capsule**

What do we already know about this issue?

*Patient delays in presentation to an emergency department (ED) are one of the main contraindications to treatment for acute ischemic strokes*.

What was the research question?

*Our objective was to identify patient and environmental factors associated with delayed presentations to the ED*.

What was the major finding of the study?

*Time of day, patient language, cerebrovascular risk factors, and location of hospital were all significantly associated with delays in presentation*.

How does this improve population health?

*Identifying barriers to prompt presentation to an ED following the onset of stroke symptoms is the first step in identifying how to best educate patients on stroke risk and recognition*.

### Outcomes

Our primary outcome was whether a patient arrived in the ED within the “presentation window” for tPA for AIS. Guidelines for the use of tPA in AIS require that it be initiated with 4.5 hours of symptom onset.^3^,^4^ The American Heart Association/American Stroke Association (AHA/ASA) guidelines further recommend that tPA be initiated within 60 minutes of arrival to the ED.^4^ Thus, patients who arrived within 3.5 hours of symptom onset were defined as having arrived within the “presentation window” in which tPA could be expected to be initiated within 4.5 hours of symptom onset.

### Data Management and Statistical Analyses

We performed all data management and statistical analysis using Statistical Analysis System (SAS) (SAS Institute, Inc., Cary, NC). Descriptive statistics were calculated for all variables. We reported continuous data as medians with interquartile ranges (IQR) and categorical variables as percentages with 95% confidence intervals (CI). A *p*-value < 0.05 was considered statistically significant. We assessed inter-rater reliability on the outcome variable using Cohen’s kappa. A random sample of 15% of cases were re-abstracted with near-perfect agreement (κ = 0.96).

We used unadjusted logistic regression to estimate the association of each patient and environmental variable with patient presentation to the ED within the treatment window. Hierarchical multivariable logistic regression was used to estimate associations between patient and environmental factors and presentation to the ED within the treatment window. We assessed effect modification between gender and chief complaint as well as language and chief complaint. Significant collinearity was identified between race and insurance as well as language and insurance; thus, we removed insurance from the final multivariable model.

### Sample Size Estimation

The parent study was powered to estimate adherence variation from an a priori-defined 95% adherence threshold.^5^ The parent study included 117 patients with AIS from each hospital, for a total 351 patients.

## RESULTS

Table 1 describes the sociodemographics, cerebrovascular comorbidities, and presenting characteristics of the 351 patients. The median time from symptom onset to presentation to the ED was 420 minutes (IQR [90–1020]) (i.e., seven hours). Only 37% of patients presented to the ED within the treatment window. For patients arriving within the treatment window, the median time from symptom onset was 60 minutes (IQR [30–120]) as compared to 840 minutes (IQR [480–2160]) for patients who arrived outside the treatment window.

Table 2 describes both the unadjusted and adjusted associations between patient and environmental variables and delayed presentations to the ED after the onset of stroke symptoms. Adjusted results show that patients who presented in the evening hours were significantly less likely to have a delayed presentation as compared to patients who presented during daytime hours (odds ratio [OR] {0.45}, 95% CI [0.3–0.8]). Speaking a language other than English (Spanish [OR {3.3}, 95% CI {1.2–8.9}] and “other” [OR {9.1}, 95% CI {1.2–71.0}]), having known cerebrovascular risk factors (>two risk factors [OR 2.4, 95% CI {1.05–5.4}] and one to two risk factors [OR {2.3}, 95% CI {1.03–5.1}]), and presenting to a rural hospital (OR [2.2], 95% CI [1.2–4.2]) were significantly associated with delayed presentation.

## DISCUSSION

Despite the significant effect of stroke on morbidity and mortality in the U.S., much of the literature for AIS focuses on the importance of minimizing treatment delays in patients who present to the ED within the tPA treatment window.^13^–^19^ As acknowledged in a statement from the AHA, the weak link in applying stroke treatments is patient delay in seeking care.^20^ Unfortunately, our results mirror those reported in the literature over the past 30 years, which show that the vast majority of patients are excluded from treatment due to delays in presentation.^20^–^28^

We identified four possible barriers to prompt presentation to an ED in our cohort: primary language, stroke risk, time of day of ED presentation, and hospital location. Speaking a primary language other than English was significantly associated with delays in presentation to the ED. Two possible explanations for our finding include differences in knowledge and recognition of stroke symptoms, and reluctance to use emergency medical services (EMS) given a language barrier.^29^,^30^ We expected patients with known stroke-risk factors to present to the ED promptly. However, we found the opposite, which contrasts with Lacy who showed no association.^31^ Given that we treated all risk factors equally in our analysis, it is possible that patients with less-obvious stroke comorbidities were unaware of their personal risk for stroke.^32^,^33^

The association of time of day and timing of ED presentation is likely explained by the effect of nocturnal onset of symptoms. Patients presenting in the morning after awakening with symptoms are almost always outside the treatment window as their last known normal time was their bedtime.^34^,^35^ While not specifically abstracted, we estimate that 12% of our cohort had “wake-up” strokes. Moreover, patients who presented in the evening hours were likely to have developed symptoms when family or co-workers were present to notice the symptoms. Lastly, it is not surprising that patients who present to a more rural hospital would have delays in presentation. While we do not have information on each patient regarding their exact distance traveled to each hospital in the study, it is reasonable to expect that patients presenting to more rural hospitals would have longer transport times than patients presenting to more urban hospitals.^36^

## LIMITATIONS

The primary limitation of this study was its use of secondary data. While these data captured the appropriate population to address our study objective, important confounders were not measured, namely EMS use and stroke severity, both of which have been shown to be associated with timing of arrival to the ED.^25^–^27^,^37^

## CONCLUSION

Important patient and environmental factors are significantly associated with delayed ED presentations following the onset of stroke symptoms. Identifying how best to educate patients on stroke risk and recognition remains critically important.

## Supplementary Information



## Figures and Tables

**Table 1:** Characteristics of patients presenting with stroke symptoms.

	Combined cohort		Inside presentation window		Outside presentation window*	
			
	%	(n)	%	(95% CI)	%	(95% CI)
Cohort	100	(351)	36.8	(32–42)	63.2	(58–68)
Time since normal (median minutes, IQR)	420.0	(90–1020)	60.0	(30–120)	840.0	(480–2160)
Sociodemographics						
Age (median, IQR)	66.0	(57–78)	69.0	(57–80)	65.0	(57–77)
Gender						
Male	49.3	(173)	50.4	(42–59)	48.6	(42–55)
Female	50.7	(178)	49.6	(41–58)	51.4	(45–58)
Race/ethnicity						
Non-Hispanic White	54.4	(191)	52.7	(44–61)	55.4	(49–62)
Hispanic	25.9	(91)	28.7	(22–37)	24.3	(19–30)
Non-Hispanic Black	16.0	(56)	14.7	(10–22)	16.7	(12–22)
Other	3.7	(13)	3.9	(2–9)	3.7	(2–7)
Language						
English	86.9	(305)	90.7	(84–95)	84.7	(79–89)
Spanish	9.1	(32)	7.0	(4–13)	10.4	(7–15)
Other	4.0	(14)	2.3	(1–7)	5.0	(3–9)
Primary insurance						
Medicare	52.1	(183)	56.6	(48–65)	49.6	(43–56)
Medicaid	8.6	(30)	5.4	(3–11)	10.4	(7–15)
Commercial	16.0	(56)	17.8	(12–25)	14.9	(11–20)
Other source	16.8	(59)	16.3	(11–24)	17.1	(13–23)
Uninsured	6.6	(23)	3.9	(2–10)	8.1	(5–14)
Patient risk and complaint						
Comorbidities						
Atrial fibrillation	12.8	(45)	13.2	(8–20)	12.6	(9–18)
Cerebrovascular disease	26.5	(93)	24.0	(13–26)	27.9	(22–34)
Congestive heart failure	5.4	(19)	7.0	(3–11)	4.5	(2–8)
Coronary artery disease	18.5	(65)	21.7	(15–30)	16.7	(12–22)
Diabetes	30.5	(107)	27.1	(15–29)	32.4	(27–39)
Hypercholesterolemia	36.8	(129)	33.3	(26–42)	38.7	(33–45)
Hypertension	72.1	(253)	71.3	(63–78)	72.5	(66–78)
Tobacco use	31.9	(112)	28.7	(22–37)	33.8	(28–40)
Chief complaint						
Typical for stroke	68.4	(240)	68.2	(60–76)	68.5	(62–74)
Associated with stroke	27.4	(96)	26.4	(20–35)	27.9	(22–34)
Other	4.3	(15)	5.4	(3–11)	3.6	(2–7)
Environmental variables						
Time of presentation						
Day (7 AM–4:59 PM)	64.1	(225)	55.0	(46–63)	69.4	(63–75)
Evening (5 PM–11:59 PM)	27.6	(97)	36.4	(29–45)	22.5	(18–28)
Night (midnight–6:59 AM)	8.3	(29)	8.5	(5–15)	8.1	(5–12)
Day of week						
Weekday (Mon 7 AM–Fri 4:59 PM)	63.0	(221)	59.7	(51–68)	64.9	(58–71)
Weekend (Fri 5 PM–Mon 6:59 AM)	37.0	(130)	40.3	(32–49)	35.1	(29–42)
Hospital location						
Rural	33.3	(117)	37.2	(29–46)	31.1	(25–37)
Suburban	33.3	(117)	39.5	(32–48)	29.7	(24–36)
Urban	33.3	(117)	23.3	(17–31)	39.2	(33–46)

*CI*, confidence interval; *IQR*, interquartile range.

* Presentation window defined as presenting in ≤ 210 minutes from onset of symptoms.

**Table 2:** Bivariate and multivariate associations for late presentation (> 3.5 hours) to emergency department after onset of stroke symptoms.

	Unadjusted		Multivariable model	
		
	OR	(95% CI)	OR	(95% CI)
Sociodemographics				
Age	0.99	(0.98–1.01)	0.98	(0.97–1.00)
Gender				
Male	Ref		Ref	
Female	1.07	(0.70–1.65)	1.06	(0.66–1.70)
Race/Ethnicity				
Non-Hispanic White	Ref		Ref	
Hispanic	0.75	(0.45–1.26)	0.58	(0.30–1.11)
Non-Hispanic Black	0.99	(0.54–1.85)	1.16	(0.57–2.34)
Other	0.95	(0.30–3.24)	0.31	(0.05–1.94)
Language				
English	Ref		Ref	
Spanish	1.51	(0.71–3.43)	3.25	(1.20–8.88)
Other	2.51	(0.77–11.3)	9.13	(1.17–71.0)
Primary insurance*				
Medicare	Ref			
Medicaid	2.33	(1.00–6.13)		
Commercial	0.82	(0.45–1.50)		
Other source	1.20	(0.66–2.21)		
Uninsured	2.01	(0.80–5.79)		
Patient risk and complaint				
Number of stroke comorbidities				
None	Ref		Ref	
1–2	1.92	(0.90–4.09)	2.3	(1.03–5.14)
> 2	2.00	(0.93–4.32)	2.4	(1.05–5.44)
Chief complaint				
Typical for stroke	Ref		Ref	
Associated with stroke	1.01	(0.62–1.65)	1.05	(0.61–1.79)
Other	0.72	(0.25–2.13)	0.67	(0.22–2.07)
Environmental variables				
Time of presentation				
Day (7 AM–4:59 PM)	Ref		Ref	
Evening (5 PM–11:59 PM)	0.54	(0.33–0.88)	0.46	(0.27–0.77)
Night (midnight–6:59 AM)	0.87	(0.40–1.98)	0.66	(0.28–1.57)
Day of week				
Weekday (Mon 7 AM–Fri 4:59 PM)	Ref		Ref	
Weekend (Fri 5 PM–Mon 6:59 AM)	0.85	(0.54–1.32)	0.88	(0.54–1.44)
Hospital location				
Urban	Ref		Ref	
Rural	1.76	(1.03–3.04)	2.23	(1.18–4.20)
Suburban	0.84	(0.50–1.41)	0.76	(0.42–1.39)

*OR*, odds ratio; *CI*, confidence interval; *Ref*, reference value.

* Multicollinearity between race and insurance, and language and insurance.
