# Supplementary material for: Barriers to Prompt Presentation to Emergency Departments in Colorado after Onset of Stroke Symptoms
Source: West J Emerg Med. 2018 Dec 5;20(2):237–43. doi: 10.5811/westjem.2018.10.38731 (PMC6404721; doi:10.5811/westjem.2018.10.38731)
Supplement: Supplementary file 1 [file wjem-20-237-s001.doc]

**____________________________________________________________________**

Patient Identifiers

MR:

Date of Admission:

Provider Identifiers

Hospital:

ED Attending:

**Step 1: Does the patient meet criteria for inclusion?**

Inclusion Criteria:

1. Age ≥ 18 YES NO
2. Hospital discharge diagnosis of Acute Ischemic Stroke YES NO

(3) ED diagnosis or initiated treatment for the above diagnoses YES NO

(4) Admission to the hospital from the ED YES NO

Exclusion Criteria:

1. Transfer from another facility YES NO

***If the patient did not meet criteria for inclusion, STOP!***

**Step 2: Review the medical record and answer the question below.**

What was the patient’s chief complaint in the ED?_________________________________________

What was the primary diagnosis in the ED? ______________________________________________

What service admitted the patient? Medicine Neurology Other ___________

What type of floor was the patient admitted to? Floor Step-down ICU

If admitted to the ICU, when did the patient leave the ICU? _________________________________

If admitted to floor or step-down, was the patient ever transferred to the ICU? YES NO

If yes 🡪 Date of transfer to ICU ___________________ Date left ICU __________________________

Did the patient die while in the hospital? YES NO

Did the patient have any of the following comorbidities?

*(review ED note, inpatient H&P, or another note from < 1 year from admission)*

coronary artery disease hypertension diabetes hyperlipidemia

cerebrovascular disease tobacco abuse atrial fibrillation

**Step 3: Review the medical record to answer the following questions.**

**If unknown, please mark “?”.**

**Acute Ischemic Stroke**

(1) When was the last time the patient was normal? __________________

(2) When was the CT done? Date _____________ Time ____________

(3) When was the CT read? Date _____________ Time ____________

(4) Did the patient receive tPA in the ED? YES NO ?

When was it given: Date _____________ Time ____________

(5) If no, did the patient have a documented contraindication to tPA? YES NO ?

*(see Table 1 below…includes presenting after 4.5 hours of symptom onset)*

(6) Did the patient or medical decision maker refuse tPA? YES NO ?

| **Table 1: Contraindications to tPA** |  |
| --- | --- |
| Active internal bleeding/peptic ulcer or bleeding diathesis  (platelets < 100,000, heparin < 48 hours w/ elevated PTT, use Coumadin with PT >15 sec)  SBP >180 (>185 stroke) or DBP >110  Major surgery or GI/GU bleed in past 3 weeks  Prior stroke or head trauma within past 3 months  Any history of intracranial bleed  Known intracranial mass or vascular abnormality  Noncompressible vascular puncture in last week  Pregnancy | Additional for AMI: suspect aortic dissection, CPR > 10 min  Additional for Stroke: Presented to ED outside the 3-4.5hr window of symptom onset, Glucose <50 or >400, suspect subarachnoid hemorrhage, seizure prior to stroke, rapidly improving symptoms, AMI in past 3 months, severe stroke (edema/mass effect/midline shift on CT OR NIHSS > 22)  Additional Contraindications tPA 3-4.5 hours in  Age > 80 years  Any use of Coumadin (regardless of PT/INR)  History of diabetes AND prior stroke |
